# Fast and robust population transfer with a Josephson qutrit via shortcut to adiabaticity

**DOI:** 10.1038/s41598-018-27697-3

**Published:** 2018-06-18

**Authors:** Zhi-Bo Feng, Xiao-Jing Lu, Run-Ying Yan, Zheng-Yin Zhao

**Affiliations:** School of Electric and Mechatronics Engineering, Xuchang University, Xuchang, 461000 China

## Abstract

We propose an effective scheme to implement fast and robust population transfer with a Josephson qutrit via shortcut to adiabaticity. Facilitated by the level-transition rule, a Λ-configuration resonant interaction can be realized between microwave drivings and the qutrit with sufficient level anharmonicity, from which we perform the reversible population transfers via invariant-based shortcut. Compared with the detuned drivings, the utilized resonant drivings shorten the transfer times significantly. Further analysis of the dependence of transfer time on Rabi couplings is helpful to experimental investigations. Thanks to the accelerated process, transfer operation is highly insensitive to noise effects. Thus the protocol could provide a promising avenue to experimentally perform fast and robust quantum operations on Josephson artificial atoms.

## Introduction

Behaving as a fundamental coherent control, quantum population transfer (QPT) is a critical issue in the context of quantum information science and state engineering^1–5^. The pursuit of QPT in an accelerated manner has been an interesting topic recently. This is mainly because target state transfer can be accomplished rapidly, which thus makes the decoherence effects on quantum operations reduced greatly. For performing the fast QPT, some novel strategies have been put forward during the past years^6–10^. Particularly, there has been a great deal of investigations on shortcuts to adiabaticity (STA), a set of methods consisting of invariant-based inverse engineering^11,12^, transitionless quantum driving^13–15^ and fast-forward scaling^16^. Compared to the adiabatic process, the STA can carry out nonadiabatically the accelerated state transfer within a much shorter time while remaining the high robustness against parameter fluctuations^17–20^. By means of a counter-diabatic driving, one can steer the rapid evolution of a system. The technique of transitionless quantum driving has been adopted widely for nonadiabatically addressing the dynamical behaviors and state engineering with different systems^21–25^. Much effort has also been invested toward the accelerated population transfers with two- and three-level systems via the invariant-based shortcut^11,26–28^.

Artificial atoms of superconducting quantum circuits are one of the most appealing candidates for quantum information processing^29–31^. By the external bias voltages and magnetic fluxes, population transfer with the artificial atoms have attracted considerable attention both theoretically and experimentally^32–38^. However, for the practical implementation of QPT, the noise-induced decoherence effects on superconducting artificial atoms are severe generally^39–41^. Isolating the considered system from the surrounding noises is a widely adopted approach to implement robust quantum operations. Alternatively, the exploration of fast QPT within a shorter time is highly preferable to diminish the decoherence effects. Based on the technique of STA, many valuable strategies for accelerating quantum operations with Josephson quantum circuits have been studied increasingly^42–45^. Very recently, an efficient protocol has been proposed for speeding up QPT in a transmon qutrit via the invariant-based shortcut^46^, in which a Λ-type qutrit was reduced into an effective two-level system after adiabatically eliminating the intermediate state in the large detuning regime. It is found that the largely-detuned drivings could lead to slower operations than that induced by the resonant drivings. In another work^47^, assisted by a counter-diabatic driving, the accelerated QPT in a Δ-type qutrit has been explored using a Josephson charge-phase quantum circuit, where three different microwave drivings need to be applied to the qutrit simultaneously.

Inspired by the above remarkable progress, we develop a promising scheme for performing fast and robust QPTs with a charge-phase qutrit via invariant-based inverse engineering. At a magic bias point, the first three levels constitute our qutrit. Allowed by the level-transition rule, we obtain a Λ-configuration resonant interaction between the qutrit and microwave drivings, containing a microwave gate voltage and a time-dependent bias flux. By the technique of invariant-based STA, we address the reversible population transfer in an accelerated way. Particularly, for speeding up transfer operations, the utilized resonant drivings in our scheme outperform the detuned drivings remarkably. The time dependence of coherent transfer on Rabi couplings could provide the experimental performance with some optimal choices. With the accessible decoherence rates, the protocol possesses high robustness due to a short operation time. So, the proposed QPT with the qutrit driven by two resonant drivings could offer a potential approach to experimentally implement fast and robust transfer operations on Josephson artificial atoms.

## System and Model

**Figure 1:**
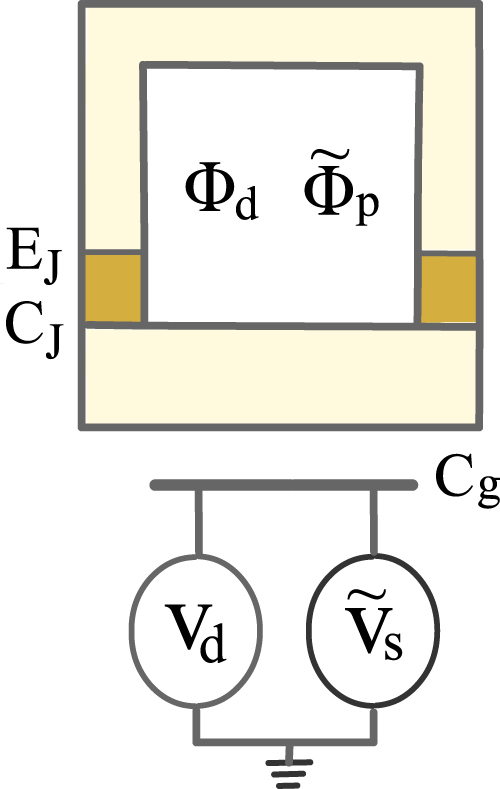
Schematic diagram of the considered artificial atom of a Josephson circuit driven by two microwave fields.

As schematically depicted in Fig. 1, a Cooper-pair box (CPB) circuit under consideration includes a superconducting box with *n* extra Cooper pairs, in which the charging energy scale of the system is *E*_*c*_. Through two symmetric Josephson junctions (with the identical coupling energies *E*_*J*_ and capacitances *C*_*J*_), the CPB is linked to a segment of a superconducting ring. In the charge-phase regime^48^, the characteristic system parameter *E*_*J*_ has the same order of magnitude as *E*_*c*_, which satisfy Δ ≫ *E*_*J*_ ~ *E*_*c*_ ≫ *k*_*B*_*T*, in which the large energy gap Δ prohibits the quasiparticle tunneling, and *k*_*B*_*T* stands for a low energy of thermal excitation. The CPB is biased by a static voltage *V*_*d*_ through a gate capacitance *C*_*g*_. Meanwhile, a static magnetic flux Φ_*d*_ through the ring aims at adjusting the effective Josephson coupling *E*_*Jd*_. An ac gate voltage ${\tilde{V}}_{s}$ is applied to the box through gate capacitance *C*_*g*_ as well, and a time-dependent flux ${\tilde{{\rm{\Phi }}}}_{p}$ threads the ring. Here the microwave drivings are used to induce the desired level transitions^46,49^, as mentioned below.

In the absence of the microwave drivings ${\tilde{V}}_{s}$ and ${\tilde{{\rm{\Phi }}}}_{p}$, the static Hamiltonian of the CPB system is given by *H*_0_ = *E*_*c*_(*n* − *n*_*d*_)^2^ − *E*_*Jd*_*cos θ*, in which *E*_*c*_ = 2*e*^2^/*C*_*t*_, with *C*_*t*_ = 2*C*_*J*_ + *C*_*g*_ being the total capacitance, and *θ* denotes the average phase difference of the two junctions, which is canonically conjugate to *n*, namely, [*θ*, *n*] = *i*. The polarized gate charge induced by *V*_*d*_ is *n*_*d*_ = *C*_*g*_*V*_*d*_/2*e*, and ${E}_{Jd}=2{E}_{J}\,\cos (\pi \frac{{{\rm{\Phi }}}_{d}}{{{\rm{\Phi }}}_{0}})$ is the effective Josephson coupling, in which Φ_0_ = *h*/2*e* indicates the flux quantum. Within the basis of Cooper-pair number states $\{|n\rangle ,|n+1\rangle \}$, the above Hamiltonian can be formally rewritten as

1 $${H}_{0}=\sum _{n}[{E}_{c}{(n-{n}_{d})}^{2}|n\rangle \langle n|-\frac{{E}_{Jd}}{2}(|n\rangle \langle n+1|+H\mathrm{.}c\mathrm{.})],$$

where the first term is the charging energy, and the second one represents the Josephson coupling.

**Figure 2:**
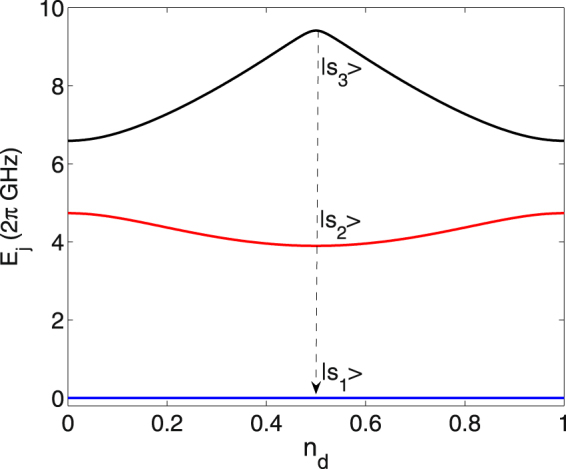
The first three eigenlevels *E*_*j*_ of the CPB system vs the gate charge *n*_*d*_, with *j* = 1, 2, and 3. The system parameters are *E*_*c*_/*h* = 3.3 GHz and *E*_*Jd*_ = 1.3*E*_*c*_. Level states $|{s}_{1}\rangle$, $|{s}_{2}\rangle$, and $|{s}_{3}\rangle$ are chosen at *n*_*d*_ = 0.5. Here *E*_1_ has been taken as the zero-energy reference.

In light of Eq. (1), we can obtain the eigenlevels and eigenstates of the static charge-phase system. With the Josephson coupling *E*_*J*_ = *E*_*c*_, we get *E*_*Jd*_ = 1.3*E*_*c*_ by adjusting Φ_*d*_. And then the first three levels *E*_*j*_ versus *n*_*d*_ are plotted in Fig. 2, with *j* = 1, 2, and 3. At a magic point of *n*_*d*_ = 0.5, we deal with three eigenstates $|{s}_{j}\rangle$, in which each state can be expressed as a superposition of Cooper-pair number states, i.e., $|{s}_{j}\rangle ={\sum }_{n}{c}_{jn}|n\rangle$, with *c*_*jn*_ being the superposition coefficients. The quantum states at the magic point are insensitive to the first-order dephasing effect, which thus contributes to prolong the decoherence time of the system^48^. Driven by the considered microwave fields, the first three levels at the magic point can be decoupled from the fourth level due to the prohibition of the level-transition rule^47^. Thus the three-level subspace $\{|{s}_{1}\rangle ,|{s}_{2}\rangle ,|{s}_{3}\rangle \}$ is selected to constitute an effective qutrit under consideration. It is found that level anharmonicity in the qutrit is enough, leading to energy spacings *ω*_32_ = (*E*_3_ − *E*_2_)/*ℏ* and *ω*_21_ = (*E*_2_ − *E*_1_)/*ℏ* far away from each other. The sufficient anharmonicity can eliminate the leakage errors induced by the coherent drivings, which is highly beneficial for performing robust population transfer with the qutrit^35,49^.

**Figure 3:**
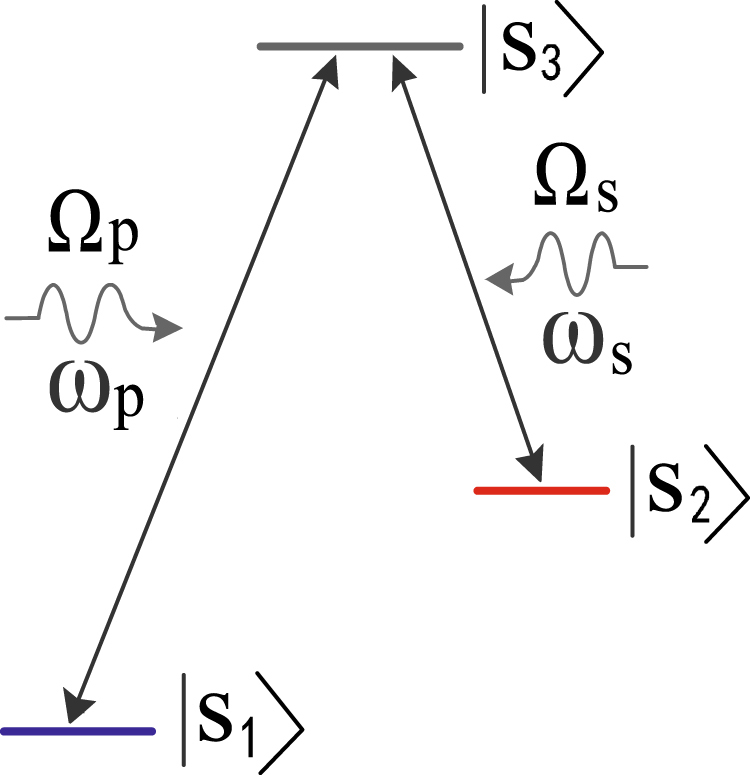
A Λ-configuration interaction between the qutrit and the microwave drivings ${\tilde{{\rm{\Phi }}}}_{{\rm{p}}}$ and ${\tilde{V}}_{s}$ with the corresponding frequency *ω*_*p*_ and *ω*_*s*_.

As shown in Fig. 3, two classical microwave drivings ${\tilde{{\rm{\Phi }}}}_{p}$ = Φ_*p*_cos(*ω*_*p*_*t*) and ${\tilde{V}}_{s}$ = *V*_*s*_cos(*ω*_*s*_*t*), acting as the corresponding pump and Stokes fields, are applied to induce the desired level couplings $|{s}_{1}\rangle \leftrightarrow |{s}_{3}\rangle$ and $|{s}_{2}\rangle \leftrightarrow |{s}_{3}\rangle$, respectively, where the microwave frequency *ω*_*p*_ (*ω*_*s*_) is resonantly matched with the transition frequency *ω*_31_ (*ω*_32_)^47^. Note that the amplitudes Φ_*p*_ and *V*_*s*_ are controllable here. Different from the previous works that induced level transitions only via electrical interactions^35,49^, the present scheme adopts both ac voltage and time-dependent bias flux. Owing to the sufficient level anharmonicity, there exists a large detuning *δ*_*s*_ = *ω*_*s*_ − *ω*_21_, and thus the ${\tilde{V}}_{s}$-induced transition between $|{s}_{1}\rangle$ and $|{s}_{2}\rangle$ vanishes nearly. Since the amplitude Φ_*p*_ (*V*_*s*_) is much smaller than Φ_*d*_ (*V*_*d*_) in our scenario, the effects of Φ_*p*_ and *V*_*s*_ on the eigenlevels can be ignored safely.

In our scheme, we treat a Λ-type interaction between the qutrit and the microwave drivings. The magnetic interaction between the CPB system and the bias flux ${\tilde{{\rm{\Phi }}}}_{p}$ reads

2 $${H}_{p}=-\frac{{E}_{Jp}}{2}\sum _{n}(|n\rangle \langle n+1|+H\mathrm{.}c\mathrm{.}),$$

which has a non-diagonal coupling form within the basis $|n\rangle$. The amplitude of ${\tilde{{\rm{\Phi }}}}_{p}$ is much smaller than Φ_0_, which yields $\cos ({\pi }\frac{{\tilde{{\rm{\Phi }}}}_{p}}{{{\rm{\Phi }}}_{0}})\approx 1$ and $\sin (\pi \frac{{\tilde{{\rm{\Phi }}}}_{p}}{{{\rm{\Phi }}}_{0}})\approx \pi \frac{{\tilde{{\rm{\Phi }}}}_{p}}{{{\rm{\Phi }}}_{0}}$. In this situation, the Josephson coupling induced by the time-dependent bias flux becomes ${E}_{Jp}=2{E}_{J}\frac{\pi {\tilde{{\rm{\Phi }}}}_{p}}{{{\rm{\Phi }}}_{0}}\,\sin (\pi \frac{{{\rm{\Phi }}}_{d}}{{{\rm{\Phi }}}_{0}})$. Facilitated by the parity-symmetry determined selection rule^50^, the magnetic interaction Hamiltonian *H*_*p*_ gives rise to the direct coupling between $|{s}_{1}\rangle$ and $|{s}_{3}\rangle$ at *n*_*d*_ = 0.5, the transition element of which is given by $\langle {s}_{1}|{H}_{p}|{s}_{3}\rangle ={{\rm{\Omega }}}_{p}\,\cos ({\omega }_{p}t)$, in which

3 $${{\rm{\Omega }}}_{p}=-\,{E}_{J}\pi \frac{{{\rm{\Phi }}}_{p}}{{{\rm{\Phi }}}_{0}}\,\sin (\pi \frac{{{\rm{\Phi }}}_{d}}{{{\rm{\Phi }}}_{0}}){O}_{13}$$

denotes the relevant Rabi coupling, and ${O}_{13}=\sum _{n,{n}^{^{\prime} },{n}^{^{\prime\prime} }}{c}_{1{n}^{^{\prime} }}^{\ast }{c}_{3{n}^{^{\prime\prime} }}\langle {n}^{^{\prime} }|(|n\rangle \langle n+1|+H.c.)|{n}^{^{\prime\prime} }\rangle$ is the overlap between $|{s}_{1}\rangle ={\sum }_{n^{\prime} }{c}_{1n^{\prime} }|n^{\prime} \rangle$ and $|{s}_{3}\rangle ={\sum }_{{n}^{^{\prime\prime} }}{c}_{3{n}^{^{\prime\prime} }}|n^{\prime\prime} \rangle$, where *n*′ and *n*′′ are Cooper-pair numbers.

The interaction Hamiltonian between the microwave pulse ${\tilde{V}}_{s}$ and the CPB system takes a diagonal form,

4 $${H}_{s}=-\,2{E}_{c}{\tilde{n}}_{s}\sum _{n}(n-{n}_{d})|n\rangle \langle n|,$$

where ${\tilde{n}}_{s}$ = *n*_*s*_cos(*ω*_*s*_*t*), with *n*_*s*_ = *C*_*g*_*V*_*s*_/2*e*. Here the fast oscillating term ${\tilde{n}}_{s}^{2}$ with a higher frequency 2*ω*_*s*_ has been omitted well under the rotation wave approximation (RWA)^35^. The ${\tilde{V}}_{s}$-induced transition matrix element between $|{s}_{2}\rangle$ and $|{s}_{3}\rangle$ takes ${t}_{23}=\langle {s}_{2}|{H}_{s}|{s}_{3}\rangle$. In terms of $|{s}_{2}\rangle ={\sum }_{n}{c}_{2n}|n\rangle$ and $|{s}_{3}\rangle ={\sum }_{n}{c}_{3n}|n\rangle$, we then have *t*_23_ = Ω_*s*_cos(*ω*_*s*_*t*), where

5 $${{\rm{\Omega }}}_{s}=-\,2{E}_{c}{O}_{23}{n}_{s}$$

indicates the Rabi coupling, with ${O}_{23}=\sum _{n}(n-{n}_{d}){c}_{2n}^{\ast }{c}_{3n}$ being the overlap between $|{s}_{2}\rangle$ and $|{s}_{3}\rangle$ at the bias point *n*_*d*_.

Within the eigenstate basis of $\{|{s}_{1}\rangle ,|{s}_{3}\rangle ,|{s}_{2}\rangle \}$, the Λ-configuration interaction under the reference frame rotating at frequencies *ω*_*p*_ and *ω*_*s*_ can be expressed as

6 $${H}_{I}=\frac{\hslash }{2}(\begin{array}{ccc}0 & {{\rm{\Omega }}}_{p} & 0\\ {{\rm{\Omega }}}_{p} & 0 & {{\rm{\Omega }}}_{s}\\ 0 & {{\rm{\Omega }}}_{s} & 0\end{array}),$$

where the RWA has been adopted and the two-photon resonance is satisfied, i.e., *ω*_31_ − *ω*_*p*_ = *ω*_32_ − *ω*_*s*_ = 0. Obviously, the Hamiltonian in Eq. (6) has a dark eigenstate with zero eigenenergy, which is a superposition of $|{s}_{1}\rangle$ and $|{s}_{2}\rangle$. Through adiabatically adjusting the Rabi couplings, population transfers can be implemented within the subspace $\{|{s}_{1}\rangle ,|{s}_{2}\rangle \}$ when the system evolves only along the dark state^1^. However, the adiabatic operations generally need long times, which are undesirable for some artificial systems with short decoherence times.

## Results

### Fast population transfer via invariant-based STA

The instantaneous eigenstates |*ψ*_*n*_〉 (with *n* = 0, ±) of the Hamiltonian in Eq. (6) are

7 $$|{\psi }_{0}(t)\rangle =(\begin{array}{c}\cos \,\theta \\ 0\\ -\sin \,\theta \end{array}),\,|{\psi }_{\pm }(t)\rangle =\frac{1}{\sqrt{2}}(\begin{array}{c}\sin \,\theta \\ \pm 1\\ \cos \,\theta \end{array}),$$

where *θ* = arc tan(Ω_*p*_/Ω_*s*_), and the corresponding eigenvalues are *E*_0_ = 0 and ${E}_{\pm }=\pm \,\hslash \sqrt{{{\rm{\Omega }}}_{p}^{2}+{{\rm{\Omega }}}_{s}^{2}}/2$, respectively. In ref.^51^, Lewis and Riesenfeld derived a useful relation between the solutions of the Schrödinger equation of a system with time-dependent Hamiltonian and the eigenstates of the corresponding invariant. Based on the invariant-based inverse engineering, we can speed up the population transfer significantly. In the following, we construct a desired dynamical invariant. The Hamiltonian in Eq. (6) can be rewritten as

8 $${{H}}_{I}(t)=\frac{\hslash }{2}[{{\rm{\Omega }}}_{p}(t){\hat{{T}}}_{x}+{{\rm{\Omega }}}_{s}(t){\hat{{T}}}_{y}],$$

where ${\hat{{T}}}_{x}$, ${\hat{{T}}}_{y}$ and ${\hat{{T}}}_{z}$ are spin-1 angular momentum operators^52^,

9 $${\hat{{T}}}_{x}=(\begin{array}{ccc}0 & 1 & 0\\ 1 & 0 & 0\\ 0 & 0 & 0\end{array}),\,{\hat{{T}}}_{y}=(\begin{array}{ccc}0 & 0 & 0\\ 0 & 0 & 1\\ 0 & 1 & 0\end{array}),\,{\hat{{T}}}_{z}=(\begin{array}{ccc}0 & 0 & -i\\ 0 & 0 & 0\\ i & 0 & 0\end{array})\mathrm{.}$$

The matrices ${\hat{{T}}}_{x}$, ${\hat{{T}}}_{y}$, and ${\hat{{T}}}_{z}$ satisfy the commutation relations

10 $$[{\hat{{T}}}_{x},{\hat{{T}}}_{y}]=i{\hat{{T}}}_{z},\,[{\hat{{T}}}_{y},{\hat{{T}}}_{z}]=i{\hat{{T}}}_{x},\,[{\hat{{T}}}_{z},{\hat{{T}}}_{x}]=i{\hat{{T}}}_{y}\mathrm{.}$$

The Hamiltonian in Eq. (6) possesses SU(2) dynamical symmetry^53^. Thus the relevant dynamical invariant, meeting the condition *dI*/*dt* ≡ ∂*I*(*t*)/∂*t* + (1/*i*ℏ)[*I*(*t*), *H*_*I*_(*t*)] = 0, can be constructed as

11 $$\begin{array}{c}I(t)=\frac{\hslash }{2}{{\rm{\Omega }}}_{0}(\cos \,\gamma \,\sin \,\beta {\hat{{T}}}_{x}+\,\cos \,\gamma \,\cos \,\beta {\hat{{T}}}_{y}+\,\sin \,\gamma {\hat{{T}}}_{z})\\ =\,\frac{\hslash }{2}{{\rm{\Omega }}}_{0}(\begin{array}{ccc}0 & \cos \,\gamma \,\sin \,\beta  & -i\,\sin \,\gamma \\ \cos \,\gamma \,\sin \,\beta  & 0 & \cos \,\gamma \,\cos \,\beta \\ i\,\sin \,\gamma  & \cos \,\gamma \,\cos \,\beta  & 0\end{array}),\end{array}$$

where Ω_0_ is an arbitrary constant with units of frequency to keep *I*(*t*) with dimensions of energy. Consequently, the eigenstates of the invariant *I*(*t*), which satisfy *I*(*t*)|*ϕ*_*n*_〉 = *λ*_*n*_|*ϕ*_*n*_〉, can be obtained as

12 $$|{\varphi }_{0}(t)\rangle =(\begin{array}{c}\cos \,\gamma \,\cos \,\beta \\ -i\,\sin \,\gamma \\ -\cos \,\gamma \,\sin \,\beta \end{array}),$$

and

13 $$|{\varphi }_{\pm }(t)\rangle =\frac{1}{\sqrt{2}}(\begin{array}{c}\sin \,\gamma \,\cos \,\beta \pm i\,\sin \,\beta \\ i\,\cos \,\gamma \\ -\sin \,\gamma \,\sin \,\beta \pm i\,\cos \,\beta \end{array}),$$

whose eigenvalues are *λ*_0_ = 0 and *λ*_±_ = ±Ω_0_/2, respectively.

According to Lewis-Riesenfeld theory^51,54,55^, the dynamics of the three-level system is generally governed by a superposition of orthonormal dynamical modes^51^,

$$|{\rm{\Psi }}(t)\rangle =\sum _{n}{C}_{n}{e}^{i{\alpha }_{n}}|{\varphi }_{n}(t)\rangle ,$$

where each *C*_*n*_ is a time-independent amplitude and the Lewis-Riesenfeld phases *α*_*n*_ are defined as

14 $${\alpha }_{n}(t)=\frac{1}{\hslash }{\int }_{0}^{t}\langle {\varphi }_{n}(t^{\prime} )|i\hslash \frac{\partial }{\partial {t}^{\text{'}}}-{H}_{I}(t^{\prime} )|{\varphi }_{n}(t^{\prime} )\rangle dt^{\prime} \mathrm{.}$$

In the case of three-level system, we have *α*_0_ = 0 and

15 $${\alpha }_{\pm }(t)=\mp {\int }_{0}^{t}[\dot{\beta }\,\sin \,\gamma +\frac{1}{2}({{\rm{\Omega }}}_{p}\,\sin \,\beta +{{\rm{\Omega }}}_{s}\,\cos \,\beta )\cos \,\gamma ]dt^{\prime} ,$$

where the dot represents a time derivative. Because of *dI*/*dt* = 0, the time-dependent parameters *γ* and *β* are related to Rabi frequencies Ω_*s*,*p*_ by the following equations,

16 $${{\rm{\Omega }}}_{s}=\mathrm{2(}\dot{\beta }\,\cot \,\gamma \,\cos \,\beta -\dot{\gamma }\,\sin \,\beta ),$$

17 $${{\rm{\Omega }}}_{p}=\mathrm{2(}\dot{\beta }\,\cot \,\gamma \,\sin \,\beta +\dot{\gamma }\,\cos \,\beta \mathrm{).}$$

Once the appropriate boundary conditions for *γ* and *β* are fixed, the Rabi frequencies Ω_*s*_ and Ω_*p*_ are determined to perform the desired population transfer from an initial state to a final one^12^.

To keep the state stationary at initial and final times, we set the boundary conditions for *β* and *γ* as follows,

18 $$\dot{\beta }\mathrm{(0)}=0,\,\dot{\beta }({t}_{f})=0,$$

19 $$\dot{\gamma }\mathrm{(0)}=\mathrm{0,}\,\dot{\gamma }({t}_{f})=0,$$

which imply Ω(0) = Ω(*t*_*f*_) = 0, meanwhile *H*_*I*_(*t*) and *I*(*t*) are commutative with each other at both the initial time *t* = 0 and final time *t* = *t*_*f*_. Only along the invariant eigenstate $|{\varphi }_{0}(t)\rangle$ in Eq. (12), the Hamiltonian *H*_*I*_(*t*) in Eq. (6) can drive an initial state |*s*_1_〉 = (1, 0, 0)^*T*^ to a target state |−*s*_2_〉 = (0, 0, −1)^*T*^ after a duration time *t*_*f*_, where the superscript *T* stands for matrix transposition. Based on Eqs (16) and (17), the boundary conditions of *γ* and *β* can be given by

20 $$\beta \mathrm{(0)}=\mathrm{0,}\,\beta ({t}_{f})=\frac{\pi }{2},$$

21 $$\gamma \mathrm{(0)}=\mathrm{0,}\,\gamma ({t}_{f})=0.$$

Now we design *β*(*t*) and *γ*(*t*) as polynomial ansatz,

22 $$\beta (t)=\sum _{j=0}^{3}{b}_{j}{t}^{j},$$

which satisfies the conditions in Eqs (18) and (20). And

23 $$\gamma (t)=\sum _{j=0}^{4}{a}_{j}{t}^{j}$$

meets the conditions in Eqs (19) and (21). Without loss of generality, we have reset a small value *ε* for the boundary conditions^12^

24 $$\gamma \mathrm{(0)}=\varepsilon ,\,\gamma ({t}_{f})=\varepsilon ,\,\gamma ({t}_{f}/\mathrm{2)}=\pi /4,$$

avoiding the infinite Rabi frequencies at the initial time *t* = 0 and final time *t* = *t*_*f*_.

In what follows, we concentrate on the physical realization of the shortcut-based population transfer with the available parameters. At the considered magic point *n*_*d*_ = 0.5, the overlap between states $|{s}_{2}\rangle$ and $|{s}_{3}\rangle$ induced by the electric interaction takes *O*_23_ = −0.468. By means of the adjustable *n*_*s*_(*t*), the Rabi frequency Ω_*s*_(*t*) is time dependent. When the amplitude is chosen as *n*_*s*_ = 5.8 × 10^−2^, the maximum value of Ω_*s*_ can reach up to ${{\rm{\Omega }}}_{s}^{(m)}/2\pi =0.18$ GHz for *E*_c_/*h* = 3.3 GHz. Similarly, with the time-dependent bias flux Φ_*p*_(*t*), Rabi coupling Ω_*p*_(*t*) can be controlled as well. With Φ_*p*_/Φ_0_ = 3.2 × 10^−2^ and Φ_*d*_/Φ_0_ ⋍ 0.275, the maximum Rabi coupling takes ${{\rm{\Omega }}}_{p}^{(m)}/2\pi =0.18$ GHz for a magnetic interaction-induced overlap *O*_13_ = −0.714.

**Figure 4:**
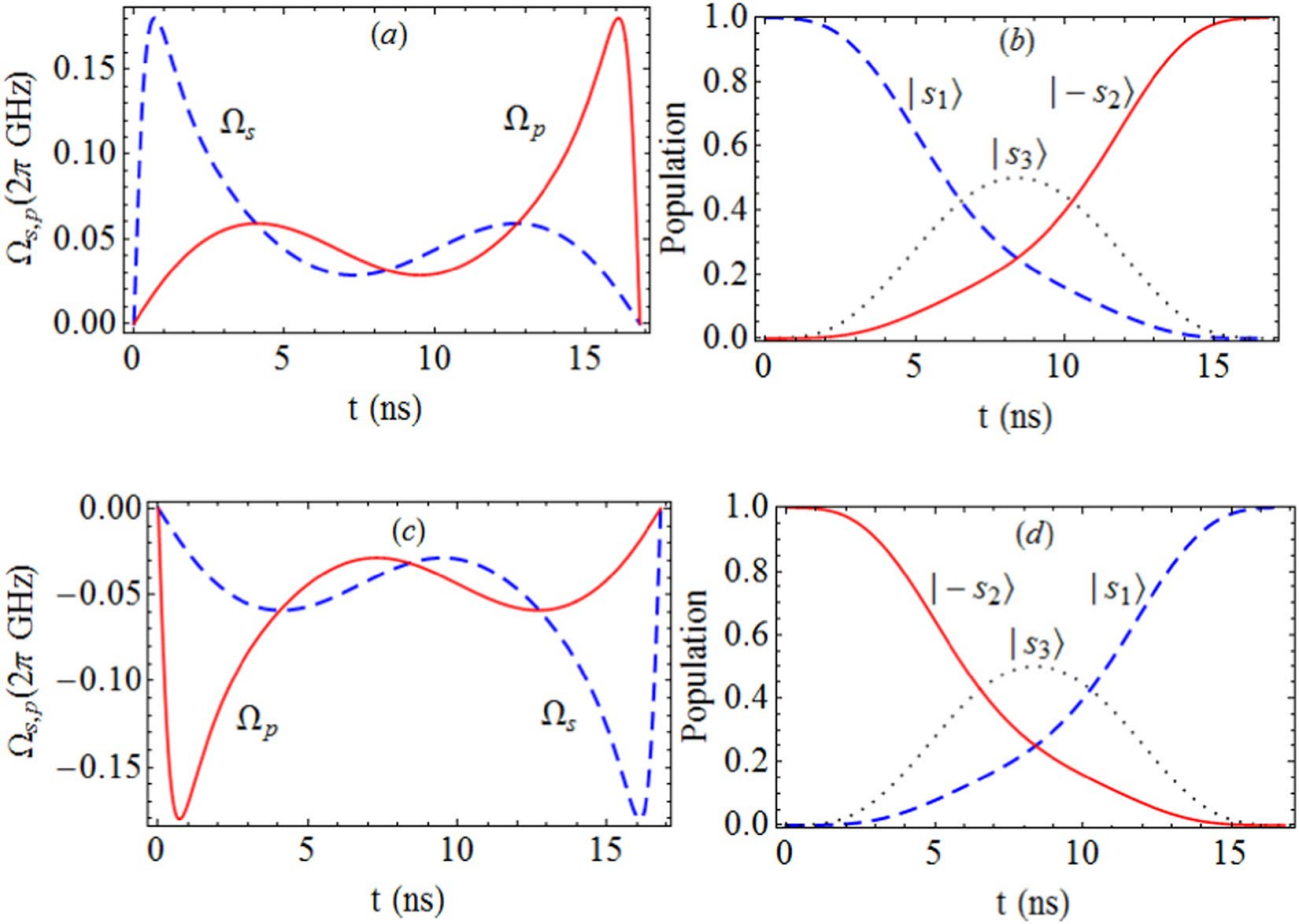
Rabi frequency Ω_*s*_ and Ω_*p*_ in (**a**), and the induced population inversion from |*s*_1_〉 to |−*s*_2_〉 via the STA in (**b**). The redesigned Rabi frequency Ω_*s*_ and Ω_*p*_ in (**c**) and the inverted population inversion from |−*s*_2_〉 to |*s*_1_〉 in (**d**). The evolution time for the reversible transfer is *t*_*f*_ = 16.8 ns, and the parameters is chosen as *ε* = 0.02.

As displayed in Fig. 4(a,b), using the polynomial ansatz in Eqs (22) and (23), we have the time-dependent Rabi couplings Ω_*s*,*p*_, by which the target population inversion from $|{s}_{1}\rangle$ to $|-{s}_{2}\rangle$ can be accomplished after a duration time *t*_*f*_ = 16.8 ns. Even with the slightly diminished couplings ${{\rm{\Omega }}}_{s,p}^{(m)}\mathrm{/2}\pi \mathrm{=0.16}$ GHz^47^, the transfer time is about 19 ns, much shorter than the adiabatic transfer time ~150 ns as discussed in ref.^47^. Here the transferred probability amplitude from the initial state $|{s}_{1}\rangle$ to target state $|-{s}_{2}\rangle$ can be formally expressed as

25 $${P}_{1\to 2}=\langle -{s}_{2}|{\varphi }_{0}({t}_{f})\rangle =\,\cos \,\varepsilon ,$$

where $|{\varphi }_{0}({t}_{f})\rangle$ is the final state at *t* = *t*_*f*_. For a chosen *ε* = 0.02 in our scheme, we get *P*_1→2_ = 99.98%, which is high enough for quantum state engineering. As an important and necessary issue for quantum information processing, the inverse population transfer from an initial state |−*s*_2_〉 to target state |*s*_1_〉 has been demonstrated in Fig. 4(d), in which the required frequencies Ω_*s*,*p*_ as functions of time are given in Fig. 4(c). As a result, the bidirectional state transfer |*s*_1_〉 ↔ |−*s*_2_〉 can be executed flexibly by adjusting the Rabi couplings.

**Figure 5:**
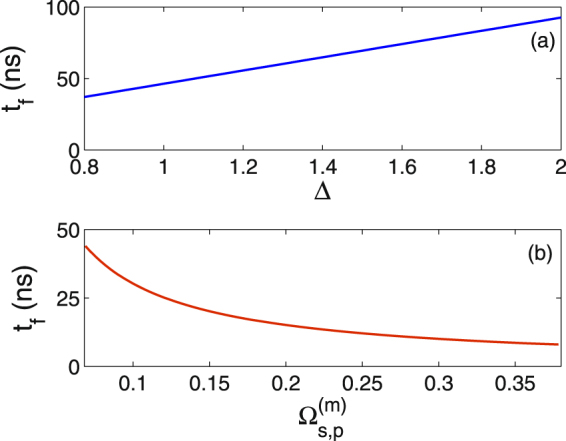
(**a**) For the maximum Rabi couplings ${{\rm{\Omega }}}_{s,p}^{(m)}/2\pi =0.18$ GHz, the needed time *t*_*f*_ for transferring |*s*_1_〉 to |−*s*_2_〉 vs Δ (in units of 2 *π* GHz). (**b**) In the resonant regime with Δ = 0, the transfer time *t*_*f*_ vs ${{\rm{\Omega }}}_{s,p}^{(m)}$ (in units of 2 *π* GHz). The parameter is taken as *ε* = 0.02.

As a distinct advantage, our protocol adopting the resonant two-photon interaction can implement the faster transfer operation, when compared with the largely-detuned drivings. For a two-photon resonance but with a common detuning Δ, the above Λ-type interaction Hamiltonian becomes

26 $${H}_{d}=\frac{\hslash }{2}(\begin{array}{ccc}0 & {{\rm{\Omega }}}_{p} & 0\\ {{\rm{\Omega }}}_{p} & 2{\rm{\Delta }} & {{\rm{\Omega }}}_{s}\\ 0 & {{\rm{\Omega }}}_{s} & 0\end{array}),$$

where Δ = *ω*_31_ − *ω*_*p*_ = *ω*_32_ − *ω*_*s*_. In the large detuning regime Δ ≫ Ω_*s*,*p*_, as mentioned in ref.^46^, level state |*s*_3_〉 is scarcely populated during the population transfer |*s*_1_〉 ↔ |*s*_2_〉. After an adiabatical elimination of the intermediate state |*s*_3_〉, a reduced two-level system within the subspace {|*s*_1_〉, |*s*_2_〉} can be obtained, whose Hamiltonian can be described by

27 $${H}_{e}=\frac{\hslash }{2}(\begin{array}{cc}-{{\rm{\Delta }}}_{e} & {{\rm{\Omega }}}_{e}\\ {{\rm{\Omega }}}_{e} & {{\rm{\Delta }}}_{e}\end{array}),$$

with an effective detuning ${{\rm{\Delta }}}_{e}=({{\rm{\Omega }}}_{p}^{2}-{{\rm{\Omega }}}_{s}^{2})/\mathrm{(4}{\rm{\Delta }})$ and Rabi coupling Ω_*e*_ = −Ω_*p*_Ω_*s*_/(2Δ). Based on the Hamiltonian in Eq. (27), the accelerated population transfers have been studied using the inverse engineering approach^46^. Here our central point of interest is the effect of detuning Δ on the transfer speed. For a state transfer from |*s*_1_〉 to |−*s*_2_〉, as indicated in Fig. 5(a), we analyze the dependence of needed time *t*_*f*_ on the detuning Δ for the utilized ${{\rm{\Omega }}}_{s,p}^{(m)}/2\pi =0.18$ GHz. It is obvious that the evolution time *t*_*f*_ increases with Δ. When Δ/2*π* = 1.0 GHz as chosen in^46^, the transfer time increases up to 46 ns. Physically, the dispersive interaction between |*s*_1_〉 and |*s*_2_〉 is built after the adiabatical elimination of the auxiliary state |*s*_3_〉, thereby the transfer process will be slowed down in contrast to the resonant case.

In addition, we address the required transfer time *t*_*f*_ versus the maximum Rabi couplings ${{\rm{\Omega }}}_{s,p}^{(m)}$, which is a crucial issue in the context of the invariant-based STA for speeding up quantum operation. Generally, *t*_*f*_ gets reduced with the increase of ${{\rm{\Omega }}}_{s,p}^{(m)}$, consistent with the Heisenberg uncertainty relation. As shown in Fig. 5(b), we numerically illustrate the dependence of *t*_*f*_ on ${{\rm{\Omega }}}_{s,p}^{(m)}$ in the resonant regime. Clearly, the suitable enhancement of ${{\rm{\Omega }}}_{s,p}^{(m)}$ with the available parameters is a feasible way for realizing the faster population transfer experimentally.

### Robustness against decoherence effects

Without dissipation effects, one could obtain an ideal population transfer with conversion probability *P*_*id*_ = 1. However, owing to the decoherence effects originating from energy relaxation and dephasing, the system evolution becomes dissipative. By adopting the standard dissipation theory, we next treat the decoherence effects on the population transfer. After tracing out the environmental degrees of freedom^56^, we have the reduced density matrix *ρ* which is associated with $|{s}_{1}\rangle$, $|{s}_{2}\rangle$, and $|{s}_{3}\rangle$. For a weak coupling between the qutrit and the environment^57^, by taking the Born-Markov approximation, the dynamical evolution of *ρ* can be characterized by the Lindblad-type master equation^58–60^

28 $$\frac{d}{dt}\rho =-\,i[{H}_{I},\rho ]+\pounds (\rho ),$$

in which the first term governs the unitary evolution subject to a Λ-type driving, and the second term^47^

$$\pounds (\rho )=\sum _{k,l=\mathrm{1,2,3}}^{k\ne l}\{{\gamma }^{(kl)}D[{\sigma }_{-}^{(kl)}]\rho +\frac{{\gamma }_{\phi }^{(kl)}}{2}D[{\sigma }_{z}^{(kl)}]\rho \}$$

contains the possible relaxations and dephasing effects caused by the noisy environment. Here *γ*^(*kl*)^ and ${\gamma }_{\phi }^{(kl)}$ are the relaxation rate and dephasing rate regarding states $|k\rangle$ and $|l\rangle$, respectively, and $D[L]\rho =\mathrm{(2}L\rho {L}^{\dagger }-{L}^{\dagger }L\rho -\rho {L}^{\dagger }L)/2$, with $L={\sigma }_{-}^{(kl)}$ and ${\sigma }_{z}^{(kl)}$. The inversion operator is defined as ${\sigma }_{-}^{(kl)}=|k\rangle \langle l|$, and Pauli operator is ${\sigma }_{z}^{(kl)}=|l\rangle \langle l|-|k\rangle \langle k|$, in which the energy levels satisfy *E*_*k*_ < *E*_*l*_.

**Figure 6:**
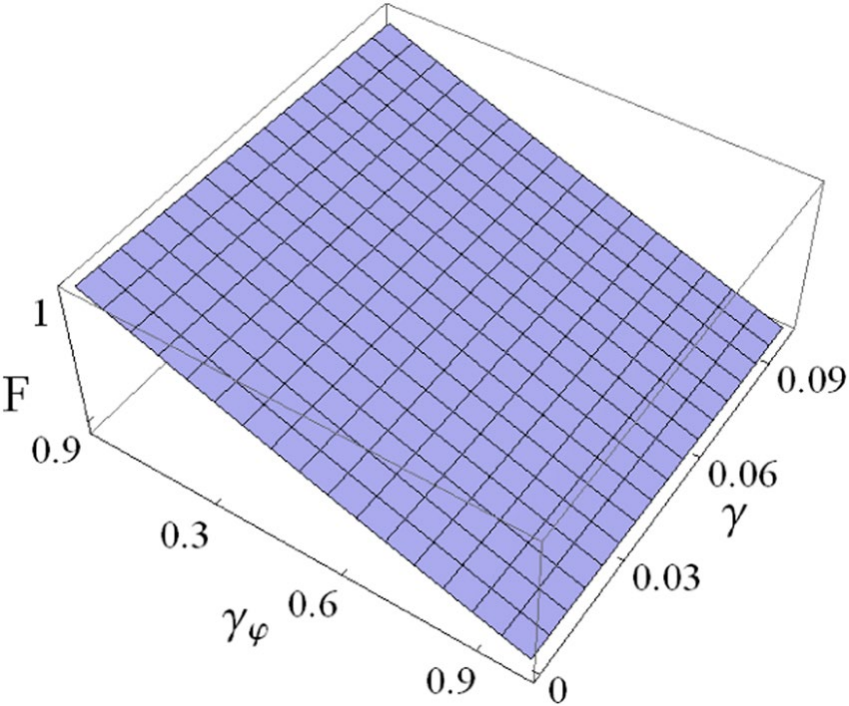
For a considered population transfer from |*s*_1_〉 to |−*s*_2_〉, fidelity *F* vs the relaxation rate *γ* and dephasing rate *γ*_*ϕ*_ (in units of 2*π* MHz).

To quantitatively represent the decoherence effects on the population transfer, we introduce a fidelity as^46^

29 $$F=\langle {\varphi }_{0}^{(i)}({t}_{f})|\rho |{\varphi }_{0}^{(i)}({t}_{f})\rangle ,$$

in which $|{\varphi }_{0}^{(i)}({t}_{f})\rangle$ is an ideal state at a given time *t* = *t*_*f*_ for a complete population inversion, and $\rho =|{\varphi }_{0}({t}_{f})\rangle \langle {\varphi }_{0}({t}_{f})|$ denotes the density matrix regarding the realistic state $|{\varphi }_{0}({t}_{f})\rangle$. Specifically, we consider the fidelity *F* of state transfer from $|{s}_{1}\rangle$ to $|-{s}_{2}\rangle$ after an evolution time *t*_*f*_ = 16.8 ns. Here assume that *γ*^(13)^ = *γ*^(23)^ = *γ*^(12)^ = *γ* and ${\gamma }_{\phi }^{\mathrm{(13)}}={\gamma }_{\phi }^{\mathrm{(23)}}={\gamma }_{\phi }^{\mathrm{(12)}}={\gamma }_{\phi }$ for simplicity. By numerically calculating Eqs (28) and (29), the fidelity *F* as a function of *γ* and *γ*_*φ*_ is displayed in Fig. 6. With the currently accessible decoherence rates *γ*/2*π* = 0.05 MHz and *γ*_*φ*_/2*π* = 0.3 MHz^61^, a robust operation having an inversion probability *P*_*in*_ = 96.42% can be performed with the qutrit. Apparently, the high robustness benefits from the rapid process by the shortcut approach.

Additionally, we justify the weak coupling condition for adopting the Lindblad-type master equation in our scenario. For the state transfer between |*s*_1_〉 and |−*s*_2_〉, as given in Fig. 4, it is numerically found that the average amplitudes of the time-dependent Rabi couplings Ω_*s*,*p*_(*t*) during the evolution processes take $\frac{1}{{t}_{f}}{\int }_{0}^{{t}_{f}}{{\rm{\Omega }}}_{s,p}(t)dt\mathrm{/2}\pi =58.4$ MHz, which are much larger than the mentioned relaxation and dephasing rates. Specifically, we have Ω_*s*_(*t*)/2*π* ≥ 3 MHz (Ω_*p*_(*t*)/2*π* ≥ 3 MHz) when *t* ∈ [5.65 × 10^−3^ ns, 16.69 ns] (*t* ∈ [0.11 ns, 16.79 ns]), see Fig. 4(a). Thereby, apart from the transient processes of initial and terminal stages, the most evolution of inducing state transfer meets the required condition well, namely, the decoherence effects are much weaker than the Rabi couplings. Moreover, with the currently chosen decoherence rates, the fidelity of state transfer between |*s*_1_〉 and |−*s*_2_〉 can reach up to 96.43% (96.42%) at *t* = 16.69 ns (16.79 ns), respectively.

## Discussion

The present strategy may have the following characteristics and advantages. (i) Compared with the adiabatic process of population transfer^1^, the shortcut-based operation has been sped up sharply and still insensitive to timing errors and parameter fluctuations, which thus have a variety of potential applications to quantum coherent control and information processing. (ii) Different from the transmon-regime quantum circuit^62^, our considered charge-phase CPB has the sufficient level anharmonicity, and then the driving-induced leakage errors can be neglected safely. The suitable level structure is very beneficial to implement the nonleaky quantum manipulation^63^. (iii) Within the Λ-type qutrit, the two-photon resonant interaction has been constructed in our scheme. By applying the invariant-based shortcut to an effective three-level system directly, we realize the faster quantum operations than that in the case of the reduced two-level system as utilized in^46^. (iv) In contrast to ref.^47^, the invariant-based engineering in the proposed protocol keeps Hamiltonian in its original form, which could provide a more straightforward way to understand the dynamical process. Only two resonant microwave drivings are needed for performing the rapid state transfers, in which the decrease in number of the drivings is highly useful to the experimental implementation. Besides, the operation time has been shortened significantly from 25 ns (with fidelity 99.81%) in ref.^47^ to 16.8 ns (with fidelity 99.98%) in the present scheme. (v) Facilitated by the direct magnetic coupling between $|{s}_{1}\rangle$ and $|{s}_{3}\rangle$, the present Λ-type qutrit is addressed at the magic point *n*_*d*_ = 0.5, which is different from the previous works^35,50^. Then the first-order dephasing effect can be removed effectively, which thus enhances the system decoherence time greatly.

In summary, we propose a promising scheme for speeding up the adiabatic population transfer in a Josephson qutrit by the technique of invariant-based STA. At the magic working point, the three lower levels constitute an effective qutrit with sufficient level anharmonicity. Based on the electric and magnetic couplings, a Λ-type resonant interaction is induced by two microwave drivings. In the resonant regime, we implement the accelerated and reversible population transfer via the invariant-based inverse engineering. As a prominent advantage, our protocol shorten the operation times significantly compared to the largely-detuned drivings. We further analyze the time dependence of state transfer on Rabi couplings, which is helpful to possible realizations. With the accessible decoherence rates, the rapid transfer operation is highly robust against the noise effects. So the protocol could offer an optimal avenue for experimentally investigating fast and robust population transfers with the Josephson artificial atoms.
